# Updated Overview of Infrared Spectroscopy Methods for Detecting Mycotoxins on Cereals (Corn, Wheat, and Barley)

**DOI:** 10.3390/toxins10010038

**Published:** 2018-01-10

**Authors:** Cecile Levasseur-Garcia

**Affiliations:** INPT, INP-Purpan, Université de Toulouse, 31076 Toulouse, France; cecile.levasseur@purpan.fr

**Keywords:** NIR, MIR, mycotoxins, *Fusarium*, infrared spectroscopy, We have updated a review of the literature dealing with infrared spectroscopy and the detection of fusariotoxins in corn, wheat, and barley. The use of infrared spectroscopic tools to detect and quantify mycotoxins does not provide the requisite regulatory precision, nevertheless, these tools can be used for sorting cereals, be they in or out of risk groups.

## Abstract

Each year, mycotoxins cause economic losses of several billion US dollars worldwide. Consequently, methods must be developed, for producers and cereal manufacturers, to detect these toxins and to comply with regulations. Chromatographic reference methods are time consuming and costly. Thus, alternative methods such as infrared spectroscopy are being increasingly developed to provide simple, rapid, and nondestructive methods to detect mycotoxins. This article reviews research conducted over the last eight years into the use of near-infrared and mid-infrared spectroscopy to monitor mycotoxins in corn, wheat, and barley. More specifically, we focus on the *Fusarium* species and on the main fusariotoxins of deoxynivalenol, zearalenone, and fumonisin B1 and B2. Quantification models are insufficiently precise to satisfy the legal requirements. Sorting models with cutoff levels are the most promising applications.

## 1. Introduction

As of 2016, wheat, barley, and maize, which are grown for both human and animal consumption, are among the most important cultivated plant species on the planet [1]. These cereals are subject to infection by the cereal pathogen *Fusarium*, which is a major concern worldwide. A *Fusarium* infection can cause plant disease and the fungus can produce fusariotoxins, which are small toxic molecules secreted as fungal secondary metabolites onto cereals and are part of the mycotoxin family [2,3,4]. These mycotoxins have a significant impact not only on public health, but also on agriculture economics and technology by reducing the yield, nutritional quality, and overall quality of the cereals [5,6,7].

The Food and Agriculture Organization (FAO) estimates that 25% of the world’s food crops are affected by mycotoxin-producing fungi. Faced with this threat, a legal framework is gradually being set up at the global level to set standards defining the maximum acceptable level of mycotoxin in foodstuffs, and enforcing such standards requires methods to detect and quantify mycotoxins [8].

The most widely used methods to monitor mycotoxins are chromatographic and immunological methods, or are based on biosensors [9,10,11,12,13]. Immunological methods, such as ELISA, can detect most of the mycotoxins, but they are mainly used for screening. The main drawbacks are the number of false positives (because of cross-reactivity and matrix-dependence) or false negatives (because of low sensitivity), in comparison with chromatographic methods. Indeed, chromatographic methods can analyze several mycotoxins at a time, with high sensitivity and selectivity, giving accurate toxins contents. Although widely used, these methods are expensive and, by requiring time-consuming extraction and clean-up steps, provide results only after a significant time lapse. Moreover, the samples are destroyed by the analysis. Cereal growers therefore require alternative methods that are simple, rapid, and that can be applied in the field, to detect mycotoxins in their products. Infrared spectroscopy has been used for many years for quality control in the food industry. In fact, the quality of cereals can be monitored by infrared spectroscopy by determining the proximate parameters (moisture, protein, oil, etc.). This technique is widely used because it is rapid, nondestructive, requires no chemicals, and is therefore eco-friendly [14].

The versatility of infrared spectrometry has led to its use in multiple applications, including soil chemistry, medicine, biology, agro-food, and cereals, and so forth. Because it probes the interactions between infrared radiation and matter, infrared spectroscopy is an appropriate tool for analyzing raw material, monitoring manufacturing processes, and validating finished products. From a theoretical point of view, this technique has no limits, provided a predictive model may be developed for the given process. The current trend consists of increasing the potential of this technique by developing new chemometric methods to create a tool adapted to a given application [15,16,17]. We distinguish four types of analyses for cereals: those related to nutritional quality (water, lipids, proteins, carbohydrate content, etc.); biological quality (germinative ability, specific purity, etc.); technological quality, and; sanitary quality (infestation by insects, pesticides, toxins, etc.). The challenge related to the presence of mycotoxins falls into the latter category. Infrared methods have limited sensitivity for contaminants. Furthermore, interpretation of infrared spectra cannot be done without the use of chemometrics, and reliable models necessitate large data set of samples (with their reference values). Despite those limitations, much research has focused on using infrared spectroscopy to monitor and analyze fusariotoxins. Mycotoxins are small molecules (MW = 700 Da) [10,13]. These toxins are found at very low concentrations, mostly parts per million (ppm), and are toxic at very low levels [13]. In fact, the level of mycotoxins is very low compared with the major seed constituents (protein, starch, etc.). Thus, analytical tools must be to detect such low concentrations; in the range of ppm (mg/mL) or a few parts per billion (ppb; ng/mL), and infrared spectroscopy currently is not sufficiently sensitive. However, changes in cereal properties such as protein, carbohydrate, or lipid content or texture are associated with changes in fungal contamination. In fact, a fungal attack damages the tissues, cells, or even molecules, and such damage is reflected in the spectral signature [18]. For example, Shenk and Workman [19] have identified spectral areas of interest related to the presence of fungi in CH_3_, CH_2_, CONH_2_, amide, starch, and cellulose structures. Mycotoxins are fungal metabolites and, like the molds that synthesize them, their distribution within the silo is heterogeneous. Thus, the sanitary quality of a given sample is not necessarily representative of the sanitary quality of the entire silo [20,21,22]. That is why some of the research reviewed deals with online infrared analysis, to avoid sampling.

This work reviews the recent research dealing with infrared spectroscopy and the detection of fusariotoxins in corn, wheat, and barley and complements a previous review [23] by summarizing the latest advances made since 2009. It relies on the reviews of McMullin et al. [24], Orina et al. [13], and Min and Cho [25], and emphasizes research involving corn, wheat, and barley.

## 2. Infrared Spectroscopy

Infrared spectroscopy reveals the interaction between infrared electromagnetic radiation (800–25,000 nm) and chemical bonds [15,26,27]. An analysis of the radiation reflected or transmitted by a sample allows one to determine the energy of the molecular overtones and of the vibrations of chemical bonds in the sample. The energy of these vibrations identifies the nature of the chemical bonds, thereby providing information about the functional groups in the molecules.

Mid-infrared spectroscopy (2500 to 10,000 nm) is widely used in analytical organic chemistry. The near-infrared (NIR) region (800 to 2500 nm or 12,500 cm^−1^ to 4000 cm^−1^) is less used because it encompasses relatively broad overtones and combinations of CH, NH, OH, and SH groups. The broad overtones and combined bands overlap, resulting in a complex spectrum with an intensity one to two orders of magnitude less than the spectral peaks in the mid-infrared. More details are available in the tutorial by Agelet and Hurburgh [28,29].

Interpreting mid-infrared spectra is straightforward because of the specificity of the absorption peaks. Conversely, interpreting NIR spectra is more complex, because they involve superpositions of spectral peaks. It also requires the use of chemometrics, which is the application of mathematical tools to obtain the maximum information possible from chemical data [30].

Developing a model requires calibrating a spectrometer with reference data obtained in the laboratory. These data (protein content, moisture content, etc.) are correlated with the infrared spectra of the samples. A predictive model is developed via five main steps [29]: (1) First, the calibration samples are selected. They must be representative of the samples that will be routinely analyzed once the model is developed and must be analyzed by using a chemical reference method (e.g., high-performance liquid chromatography or gas chromatography mass spectroscopy) [31]. The range of the “analyte” content should be covered by the range of the calibration samples. The samples are generally divided randomly into two sets: a calibration set and a validation set (about 75% and 25%, respectively) [32,33]; (2) The infrared spectra of the reference samples are then acquired either by reflection- or transmission-mode spectroscopy. In reflection mode, the spectrometer detects the intensity of the light reflected by the sample, whereas in transmission mode, the spectrometer records the intensity of light transmitted through the sample; (3) Mathematical preprocessing is applied to eliminate baseline noise and drifts; (4) A model is developed to establish a correlation between spectral values and reference values; (5) Finally, the model is validated. Once the model is developed and validated, it can be used routinely. In the following paragraphs, we provide more details about this process.

### 2.1. Spectral-Data Preprocessing

Spectral-data preprocessing [29] refers to the mathematical manipulation of raw spectral data and is used to suppress or reduce intensity variations related to factors that should not be considered in the model. The right choice of preprocessing may be critical to the development of the model. Different preprocessing methods are commonly used in infrared spectroscopy: derivative [34,35], smoothing [36], detrending (which is often used after a standard normal variation [35,37]), multiplicative scatter correction [38], and so on. Derivatives are most commonly used in mycotoxin studies [39,40,41,42].

### 2.2. Types of Regression Used to Develop Prediction Model

Two main chemometric methods are used in infrared spectroscopy: classification methods, and regression methods [43].

Classification models allow us to classify the samples into groups, called classes, based on their distinguishing spectral features. Two approaches to classification exist: supervised and unsupervised. In unsupervised classification, the spectral similarities and dissimilarities of the samples are used to create groups, whereas in supervised classification, group membership is defined at the beginning of the modeling (discriminant analysis) [30,44].

Regression methods are used to link spectra to chemical values and include linear and nonlinear methods. The three best-known linear-regression methods are multiple linear regression, principal component regression, and partial least squares regression (PLS). PLS is the most widely used method in infrared spectroscopy [29], and particularly in works concerning mycotoxins [41,45,46,47,48,49,50]. It was introduced by Wold in 1966 and popularized by Martens in the early 1980s [51]. This method consists of establishing a regression of the variable to be predicted as a function of latent variables, which are linear combinations of the original predictive variables (i.e., wavelengths). They are determined by considering the original variables and the variable to be predicted and minimizing the sum of the squares of the residuals [44]. The most used nonlinear method is the artificial neural network (ANN), which was developed in the late 1980s and early 1990s [52]. The principle of ANNs is analogous to that of biological neurons; it consists of connected neurons, each of which carries out a single task and communicates the result to one or more neuron by applying precise rules [44].

### 2.3. Validation and Performance of Model

The model’s ability to accurately predict the characteristics of new samples is ideally determined by applying the model to a set of samples that was not used to develop the model. Such a procedure constitutes an external validation of a test set. Sometimes the dataset is not sufficiently large to subdivide it into two sets, so the cross-validation method is used.

The performance of a model is evaluated mainly by the coefficient of determination *r*^2^ and the standard error of calibration (SEC) (see Table 1). The latter is based on the residuals, that is, the differences between the predicted values and the actual values of the *n* samples of the calibration set. The residuals represent the information contained in the data of the *n* reference samples which are not explained by the model [36]. The full validation of the model involves the study of the validation set. The samples in this set are used to test the predictive quality of the model by calculating the standard error of prediction (SEP) the prediction deviation, and the root mean square error of prediction (RMSEP). For cross-validation, the population of the calibration set is divided into *t* subgroups. A calibration equation is determined based on (*t* − 1) groups and then validated on the remaining group. This operation is resumed by permutation over the other subgroups. The calculated standard deviation is the standard error of cross-validation (SECV), which is often optimistic [29]. The last performance metric to report is the relative prediction determinant (RPD), which represents the ability of the model to predict unknown samples, considering the variability of the training set. Williams [53] provides RPD thresholds to give an idea of the potential for applying calibrations to new samples: if the RPD is less than 2.3, the model cannot be used, whereas if it exceeds 8, the model can be used without hesitation.

The largest *r*^2^ corresponds to the best model. In addition, the best model has the smallest *SEP_c_* [29,54].

The brief outline above describes a method to develop a predictive model based on infrared spectroscopy. These instructions are valid regardless of the type of spectrometer used [29]. In general, a spectrometer is made of several parts: a light source; a wavelength-selection system; a signal-detection system; a signal-processing system, and; an apparatus for positioning the sample [55].

## 3. Using Infrared Spectroscopy to Quantify Fusariotoxins in Corn, Wheat, and Barley

A tool to quantify mycotoxins in these cereals is of interest only if it is sufficiently sensitive and specific with respect to the maximum permissible levels for mycotoxins. Toxins should be detected, but with a low false-negative rate, which minimizes the risk of cereal contamination in the food chain [21].

The first applications of infrared spectroscopy to the analysis of microorganisms date from the 1950s [56]. In the 1980s, Fraenkel et al. [57] and Davies et al. [58] published their first work on the use of NIR spectroscopy to detect fungal contamination. Various studies have been carried out since the 1990s [23]. This work compiles the studies carried out since 2009 (Table 2).

The referenced studies target deoxynivalenol (14/19), fumonisins (4/19), zearalenone (2/19), fungi (2/19) and nivalenol (1/19). Most of the work therefore concerns DON and Fusarium head blight. This work includes quantification models or grading models in batches of toxin levels. For DON, quantification models have variable performances (Tibola et al., 2010; Dvoracek et al., 2012; Balut et al., 2013; Bezdekova and Bradacova, 2013; De Girolamo et al., 2014; Jin et al., 2014, Miedaner et al., 2015). Tibola et al. (2010) [45] worked on wheat grains naturally contaminated with *Fusarium graminearum* and DON (196 samples). The wheat samples were analyzed as whole grains (125 g), and then crushed and sieved to obtain diameters less than 1.0 mm. Reference analyses were done by combined liquid chromatography and mass spectrometry (LC-MSMS). They developed a model for predicting DON in whole-grain (crushed) grades with *r*^2^ = 0.89 (0.91). Dvoracek et al. (2012) [46] worked on whole-wheat samples to determine their DON content. They conclude that the combination of two tools, a discriminant analysis (DA) followed by a PLS regression, improves the accuracy of the models. The quality of the models depends strongly on the choice of the class limits, in terms of DON contents. Balut et al. (2013) investigated ways to predict DON and FKD levels in wheat [62]. The correlation between *Fusarium*-damaged kernels (FDKs) and FDKs predicted by NIR spectroscopy is 0.7 and 0.73, respectively, and the correlation between DON and DON predicted by NIR spectroscopy is 0.56 and 0.63 for the years 2010 and 2011, respectively. Bezdekova and Bradacova [41] applied FT-NIR to barley to determine DON and NIV content. Barley was not only inoculated artificially, but also naturally contaminated. De Girolamo et al. [49] used FT-NIR to study DON in wheat samples. The authors propose classification models that yield an interesting percent of correct classification (75–90%). Jin et al. (2014) [63] conducted a similar study with artificially inoculated wheat samples. The authors found a significant correlation between the GC-MS DON levels and the predictions by their infrared model (*r*^2^ = 0.46, *p* < 0.001), as well as a significant correlation between the number of FDKs evaluated visually and estimated by infrared spectroscopy (*r*^2^ = 0.52, *p* < 0.001). The authors explain that a bias exists in this study because the DON content determined by GC-MS was for bulk samples, whereas the spectra were collected from individual grains. Miedaner et al. [65] worked with samples of corn artificially inoculated with *Fusarium graminearum*. They predicted DON levels by using NIR spectra and obtained *r* = 0.80. No information is given on model errors.

DON assay quantification models are poor at predicting a specific grade, especially with respect to regulatory grades. However, these models can be used to classify grain lots according to a level of toxin contamination [40,41,49,64,66]. Peiris et al. (2009, 2010) [39,40] used a grain-to-grain method to identify wheat grains infected with *Fusarium* and to predict DON levels. Their findings indicate that it is possible to predict the DON concentration when it exceeds 60 ppm [39,40]. Kautzman et al. [64] developed a model based on an indirect indication of the presence of mycotoxins; namely, the crude protein level. By sorting grains based on this index and classifying them into different fractions, they reduced the DON content and the percent of FDKs (for the best batches). Levasseur-Garcia and Kleiber (2015) [50] proposed different models for identifying maize grain contaminated with DON or with fumonisins (FUM). Based on the European detection limits, the models provide correct identification at a rate of 60% and 84%, respectively, in external validation. Kos et al. [66] worked on DON in corn samples. They used a bagged decision tree classification, focusing on a spectral window corresponding to 800–1800 cm^−1^. This window contains information related to carbohydrates and proteins. The rate of proper cross-validation ranking varies between 79% and 85%, depending on the DON limit used to create the classes. Sieger et al. [67] proposed a new on-site mycotoxin analysis by combining mid-infrared tunable quantum cascade laser spectroscopy (QCL) with GaAs/AlGaAs thin-film waveguides to classify deoxynivalenol-contaminated maize and wheat samples, and aflatoxin B_1_ affected peanuts at European Union (EU) regulatory limits of 1250 μg kg^−1^ and 8 μg kg^−1^, respectively. Very few techniques are available for on-site analysis. This recent work therefore offers a very interesting opportunity for field use, thanks to miniaturization [68].

The work on fumonisins draws the same conclusions [42,47,48,50]. The best models have screening ability with thresholds corresponding to legal limits. Gaspardo et al. [47] propose a model that uses Fourier-transform NIR (FT-NIR) spectroscopy to predict FB1 and FB2 levels in corn samples. The model has a low RPD value (1.2), even for screening purposes [49,69]. Della Riccia and Del Zotto [48] developed a PLS model to predict the fumonisin levels of maize samples. They propose predictive models that can be used for classification, emphasizing the 4 mg/kg threshold stipulated by EU regulation. Stasiewicz et al. [42] tested a low-cost multispectral tool, combining both the visible and the NIR to sort fumonisin-contaminated maize grains. By using two-stage sorting, they spread the grains with a success percent close to 80%.

The work to identify levels of infection by several fungi have good performances investigated *Fusarium*-infected corn kernels [59]. They combined visible and NIR spectroscopy and used various classification approaches such as the soft independent modeling of class analogy (SIMCA) and neural networks. The spectrum of each grain was collected from both the germ side and the opposite side, with the best results coming from the side opposite the germ. Healthy and infected grains are properly graded at a rate of 99.3% and 98.7%, respectively. These results were obtained from a test set. Tallada et al. (2011) applied NIR spectroscopy (904–1685 nm) to single kernels to identify maize grain infected to varying degree with eight fungal strains. They used linear discriminant analysis (LDA) or ANNs. The objective was to discriminate between healthy and diseased grains. The ANN (LDA) model correctly discriminated 84% (89%) of healthy grains and 83% (79%) of infected grains [60].

Moreover, as mycotoxins and fungi cannot be directly detected in the grain matrix, because of the lack of sensitivity of infrared devices, indirect information of contamination is used in models. The contamination is assessed by detecting alterations of the grains. Indeed, different authors have identified spectral zones that indicate grain alterations due to fungal attack or to the presence of mycotoxins [40,66,70,71]: the changes are related to carbohydrate content (900–1200 cm^−1^) and proteins (amide bands I and II, 1200–1750 cm^−1^).

Another point concerns methods for the simultaneous analysis of several of mycotoxins [72]. This is well achieved by LC-MS/MS (liquid chromatography with tandem mass spectrometry), but in the current state of research, no studies based on an infrared tool report a multi-toxin model. This is probably a research direction for the years to come.

In a global way, the use of the infrared tool in toxin detection is an integral part of quality control of agricultural products, and monitoring of the resources. Mycotoxins could be part of a multi-parametric analysis of cereals, in seconds, with no sample preparation [73].

## 4. Conclusions

In recent years, several studies have investigated the use of spectroscopic methods to detect mycotoxins in grains. These methods are fast, inexpensive, nondestructive, and, once the model is developed, require little sample preparation and few well-trained technicians.

However, the use of infrared spectroscopic tools to detect and quantify mycotoxins does not provide the requisite regulatory precision [24]. The large standard errors show that, because of the sensitivity limits of the devices, these quantization models are insufficiently precise to satisfy the legal requirements [13,24,74].

Thus, mycotoxin detection capabilities need to be improved. Hopefully, the recent and continuous improvements in mathematical preprocessing will increase the sensitivity and precision of the models. Note that these tools can be still used for sorting food products, be they in or out of risk groups [14,75,76,77], which would allow “distinguishing, sorting, screening and monitoring” with cutoff levels [78]. Because mycotoxin-contaminated products are not evenly distributed within a given lot, large volumes must be scanned to increase the margin of safety. The most promising applications might be in-line single-kernel analyses, which would promote the production of commercial foodstuffs (feed, food, baby food) with optimal quality from any grade of raw material. All these methods detect a change in the grains related to the presence of the fungus [23,64].

This review thus highlights some of the developments over the past eight years in the detection of fusariotoxins to give an overview of the current state of the art for applying infrared spectroscopic methods to this field and to outline the most promising methods. Future work will require working on masked mycotoxins, whose importance is discussed in the review of Dall’Asta and Battilani [79]. The use of other spectroscopic techniques should also be developed; for example, Raman spectroscopy [80] or hyperspectral imaging techniques [81,82,83,84].

## Figures and Tables

**Table 1:** Performance Criteria for Validated Model [29,36,54].

*k* = number of factors, *y_i_* = *n* measured values, ${\hat{y}}_{i}=n$ predicted values $\sum d_{i}=\sum_{i=1}^{n}(y_{i}-{\hat{y}}_{i})\;\mathrm{et}\;\sum x\;=\;\sum_{i=1}^{n}x$		
***r*^2^**	Determination coefficient	$r^{2}=\frac{\sum {\left({\hat{y}}_{i}-\bar{y}\right)}^{2}}{\sum (y_{i}-\bar{y}{)}^{2}}$
**Bias (same units as reference value)**	Bias	$Bias=\frac{\sum d_{i}}{n}$
**SEC (same units as reference value)**	Standard Error of Calibration	$SEC=\frac{\sum {\left(d_{i}\right)}^{2}}{n-k-1}$
**SEPc (same units as reference value)**	Standard Error of Prediction (corrected by the bias)	$SEP_{c}=\;\sqrt{\frac{\left(d_{i}-bias\right)²}{n-1}}$
**RMSEP (same units as reference value)**	Root Mean Square Error of Prediction	$RMSEP=\sqrt{\frac{\sum {d_{i}}^{2}}{n}}$
**RPD**	Ratio of Performance to Deviation	$RPD=\frac{StdError_{ref}}{SEP_{c}}$

**Table 2:** Summary of studies of infrared spectroscopy applied to quantification of mycotoxins in barley, corn, and wheat. Deoxynivalenol (DON); Zearalenone (ZON); partial least squares regression (PLS); near-infrared (NIR); standard error of cross-validation (SECV); linear discriminant analysis (LDA); liquid chromatography and mass spectrometry (LC-MSMS); *Fusarium*-damaged kernels (FDKs).

Mycotoxin or Fungi	Crop/Number of Samples/Sample Preparation	Spectral Range	Performance and Characteristic Wavelengths	Reference
DON	Wheat: 30 kernels artificially inoculated/*Single kernel*	NIR 950–1650 nm	DON band absorption: 1408 nm, 1904 nm, 1919 nm Differences at 1204 nm, 1365 nm and 1700 nm, attributed to changes in food reserves such as starches, proteins, and lipids.	Peiris et al. (2009) [39]
Fusarium-damaged kernels	Corn: 600 spectra in training set and 300 spectra in test set/*Single kernel (spectra collected on germ side and on other side of grain)*	NIR 400–2498 nm	SIMCA classifier or Probabilistic Neural Network: best results: healthy grains well classified = 99.3%, 98.7% for infected grains Grain position (germ side or other side) is a significant factor for disease detection	Draganova et al. (2010) [59]
DON—Fusarium-damaged kernels	Wheat/*single kernels*		Prediction of DON levels in kernels having > 60 ppm DON : sorting	Peiris et al. (2010) [40]
DON—ZON	Wheat: 196 samples for DON, 120 samples for ZON/*Whole and milled grains*	NIR 400–2500 nm	Whole kernels: DON(LC-MSMS)—DON(IR): *r*^2^ = 0.89 SECV = 612.05 µg kg^−1^ Milled kernels: DON(LC-MSMS)—DON(IR): *r*^2^ = 0.91 SECV = 578.33 µg kg^−1^ Whole kernels: ZON(LC-MSMS)—ZON(IR): *r*^2^ = 0.86 SECV = 254.29 µg kg^−1^ Milled kernels: ZON(LC-MSMS)—ZON(IR): *r*^2^ = 0.87 SECV = 231.85 µg kg^−1^	Tibola et al. (2010) [45]
Aspergillus flavus, Bipolaris zeicola, Diplodia maydis, Fusarium oxysporum, Penicillium oxalicum, Penicillium funiculosum, Trichoderma harzianum	Corn: 864 inoculated *single kernels* 0 to 100% infected	NIR 904–1685 nm	All levels of infection: LDA accuracy: 89% on control, 79% on infected MLP accuracy: 84% on control, 83% on infected False negatives: caused by inclusion of asymptomatic kernels	Tallada et al. (2011) [60]
DON	399 wheat samples—*whole grains* artificially infected	FT-NIR 10,000–400 cm^−1^	Reference = ELISA PLS 0 < DON < 92 mg/kg: *r* = 0.94; SEP = 6.23 mg/kg; RPD = 3.02 0 < DON < 30 mg/kg: *r* = 0.92; SEP = 2.43 mg/kg; RPD = 2.60 PLS-DA: improvement from the best PLS model: *r* = 0.92, SEP = 2.35 mg/kg Identification of two spectral regions (1390–1770 nm and 1880–2070 nm)	Dvoracek et al. (2012) [46]
Fumonisins B1 and B2	Corn *milled grains*: 168 samples	FT-NIR 650–2500 nm	PLS: *r*^2^ = 0.964, SEC = 0.433 mg/kg, SEP = 0.839 mg/kg, RPD = 1.2	Gaspardo et al. (2012) [47]
DON—Fusarium-damaged kernels	Wheat *Grains were dissected, and each section was pressed to the ATR diamond crystal*	FT-MIR 4000–380 cm^−1^	Marked differences in absorption patterns between sound and fusarium damaged pericarp and germ spectra: shift 1035 cm^−1^ and increased absorptions at 1160, 1203, 1313, and 1375 cm^−1^ (influence of DON and fungi on wheat matrix)	Peiris et al. (2012) [61]
DON—Fusarium-damaged kernels	Wheat *Whole grains?*	NIR 950–1650 nm	FDK-FDKNIR: *r* = 0.70 (2010) and 0.73 (2011) DON-DONNIR: *r* = 0.56 (2010) and 0.63 (2011) Differences due to changes in carbohydrate, lipid, protein, and DON levels, and physical properties of the kernels	Balut et al. (2013) [62]
DON—NIV	Barley: 200 spectra—cross-validation *Milled grains*	NIR 12,000–4000 cm^−1^	DON-DONNIR: *r* = 0.875 rcrossval = 0.513 RMSEC = 0.147 e3, RMSECV = 0.268 e3RMSEP = 0.399 e3 NIV-NIVNIR: *r* = 0.828 rcrossval = 0.744 RMSEC = 0.310 e3 RMSECV = 0.371 e3 RMSEP = 0.433 e3 models applicable only for detection of highly contaminated grain lots	Bezdekova and Bradacova (2013) [41]
Fumonisins	Corn *Milled grains*	FT-NIR 650–2500 nm	PLS HPLC *r*^2^ = 0.995; SEC = 0.232, *r*^2^ = 0.908; SEP = 0.933Evaluation of the screening and classification ability with thresholds corresponding to legal limits	Della Riccia and Del Zotto (2013) [48]
DON	Wheat 464 samples *Milled grains*	FT-NIR 10,000–4000 cm^−1^	PLS traces < DON < 16,000 µg/kg RMSEP = 1977 µg/kg ≥ poor ability LDA 3 classes (DON ≤ 1000 µg/kg)-1000 < DON < 2500 µg/kg-DON > 2000 µg/kg) 75–90% accuracy	De Girolamo et al. (2014) [49]
DON—Fusarium-damaged kernels	Wheat: 291 inoculated *single kernels* DON levels: 0.49 to 29.25 mg/kg	NIR	291 samples for FDK estimation 148 samples for DON estimation DON(GC-MS)-DON(IR): *r*^2^ = 0.46, *p* < 0.001 FDK(visual): FDK(IR): (*r*^2^ = 0.52, *p* < 0.001).	Jin et al. (2014) [63]
DON—Fusarium-damaged kernels	Wheat/*Single kernel*	NIR 1100–1700 nm	Creation of several lots of varying quality (FDK et DON), based on the crude protein.	Kautzman et al. (2015) [64]
DON and fumonisins	381 samples for DON 511 samples for FUM/*Whole grains*	NIR 400–2498 nm	Reference: HPLC MS discriminant analysis Accuracies from 60 to 84% with external validation	Levasseur-Garcia and Kleiber (2015) [50]
DON—ZON	Corn artificially inoculated 9 < DON < 920 mg/kg *Milled grains*	NIR	*r*(DON/DON immunotest) = 0.80	Miedaner et al. (2015) [65]
DON	110 corn samples (naturally and artificially infected) *Milled grains—100–250 µm sieve fraction*	MIR 4000–575 cm^−1^	Carbohydrate (1000 cm^−1^) and protein (1500 cm^−1^)-related vibrations ≥ spectral window used for modelling: 1800–800 cm^−1^ Classification threshold (cross-validation): 1750 µg/kg: overall classification accuracy = 79% 500 µg/kg: overall classification accuracy = 85%	Kos et al. (2016) [66]
DON	Corn (24 samples), wheat *Grains extracts*	MIR 1820–1560 cm^−1^	Alterations of the sample matrix caused by fungal infection: 1655, 1710, 1740 cm^−1^ ≥ Classification of grain	Sieger et al. (2017) [67]
Fumonisins	Corn (453 grains) *Single kernels*	Multispectral VIS-NIR	First round: 470, 527, 624, 850, 880, 910, 940, 1070 nm second round: 910, 940, 970, 1050, 1070, 1200, 1300, 1450, 1550 nm LDA ≥ maximum cross-validation sensitivity (77%) and sensibility (83%) to reject corn kernels with fumonisin >1000 ng/g	Stasiewicz et al. (2017) [42]

## Abbreviations

ANN	Artificial neural networks
ATR	Attenuated total reflection
DA	Discriminant analysis
DON	Deoxynivalenol
FDK	Fusarium damaged kernel
FT-NIR	Fourier transform near infrared spectroscopy
FUM	Fumonisins
LC-MS	Liquid chromatography-mass spectrometry
KNN	K-nearest neighbor classification
LDA	Linear discriminant analysis
MIR	Mid-infrared wavelength
MPL	Multilayer perceptron neural network
NIR	Near-infrared wavelength
PCA	Principal component analysis
PCR	Polymerase chain reaction
PLS	Partial least squares regression
PLSDA	Partial least squares discriminant analysis
SVM	Support vector machines
VIS	Visible wavelength
ZON	Zearalenone
